# Chiral Pseudo-*D*_6h_ Dy(III) Single-Molecule Magnet Based on a Hexaaza Macrocycle

**DOI:** 10.3390/molecules30092043

**Published:** 2025-05-03

**Authors:** Jia-Hui Liu, Yi-Shu Jin, Jinkui Tang, Cai-Ming Liu, Yi-Quan Zhang, Hui-Zhong Kou

**Affiliations:** 1 Engineering Research Center of Advanced Rare Earth Materials (Ministry of Education), Department of Chemistry, Tsinghua University, Beijing 100084, China; liujh22@mails.tsinghua.edu.cn (J.-H.L.); jysthu@126.com (Y.-S.J.); 2 State Key Laboratory of Rare Earth Resource Utilization, Changchun Institute of Applied Chemistry, Chinese Academy of Sciences, Renmin Street 5625, Changchun 130022, China; 3 Beijing National Laboratory for Molecular Sciences, Center for Molecular Science, Institute of Chemistry, Chinese Academy of Sciences, Beijing 100190, China; cmliu@iccas.ac.cn; 4 Ministry of Education Key Laboratory of NSLSCS, School of Physical Science and Technology, Nanjing Normal University, Nanjing 210023, China

**Keywords:** chirality, single-molecule magnet, hexaaza macrocycle, Dy, *D*_6h_

## Abstract

A mononuclear complex [Dy(phenN_6_)(HL′)_2_]PF_6_·CH_2_Cl_2_ (H_2_L′ = *R*/*S*-1,1′-binaphthyl-2,2′-diphenol) with local *D*_6h_ symmetry was synthesized. Structural determination shows that Dy^3+^ was encapsulated within the coordination cavity of the neutral hexaaza macrocyclic ligand phenN_6_, forming a non-planar coordination environment. The axial positions are occupied by two phenoxy groups of binaphthol in the *trans* form. The local geometry of Dy^3+^ closely resembles a regular hexagonal bipyramid *D*_6h_ configuration. The axial Dy-O_phenoxy_ distances are 2.189(5) and 2.145(5) Å, respectively, while the Dy-N bond lengths in the equatorial plane are in the range of 2.524(7)–2.717(5) Å. The axial O_phthalmoxy_-Dy-O_phthalmoxy_ bond angle is 162.91(17)°, which deviates from the ideal linearity. Under the excitation at 320 nm, the complex exhibits a characteristic emission peak at 360 nm, corresponding to the naphthalene ring. The AC susceptibility measurements under an applied DC field of 1800 Oe show distinct temperature-dependent and frequency-dependent AC magnetic susceptibility, typical of single-molecule magnetic behavior. The Cole–Cole plot in the temperature range of 6.0–28.0 K was fitted using a model incorporating Orbach and Raman relaxation mechanisms, giving an effective energy barrier of *U*_eff_ = 300.2 K. Theoretical calculations on complex **1** reveal that the magnetization relaxation proceeds through the first excited Kramers doublets with a calculated magnetization blocking barrier of 404.1 cm^−1^ (581.4 K).

## 1. Introduction

Single-molecule magnets (SMMs), integrating slow magnetization relaxation and quantum tunneling effects, are potentially utilized in the fabrication of nanoscale display devices and high-density data storage media, as well as quantum computing [1,2,3,4]. However, the operating thresholds of SMMs are predominantly confined to extreme cryogenic regimes, where the magnitude of the anisotropic barrier and the blocking temperature are critical factors in determining their ambient-temperature functions [5,6,7,8]. Lanthanide-based single-molecule magnets (Ln-SMMs) have emerged as highly promising candidates for advancing high-performance magnetic materials, taking advantage of the enhanced magnetic anisotropy and large magnetic moments inherent to rare-earth elements [9,10,11,12]. The multi-functionality of lanthanides and the rational selection of functional ligands enables the development of intelligent materials for practical applications, such as luminescent, mechanochromic, and photo-responsive Ln-SMMs [13,14,15,16].

The performance of Ln-SMMs fundamentally depends on increasing magnetic anisotropy while suppressing the quantum tunneling interference in relaxation dynamics [17,18,19]. The magnitude of magnetic anisotropy in these systems is predominantly governed by two competing factors: spin–orbit coupling (SOC) strength and electrostatic crystal field effects [20,21,22]. Weak isotropic equatorial coordination minimizes 4f electron cloud repulsion within the equatorial plane, which is beneficial for SMM properties. In contrast, the axial coordination of strong-field ligands, e.g., phenoxide, alkoxide, or siloxide, maximizes axial magnetic anisotropy, which is conducive to SMMs [23,24]. Quantum tunneling and Raman relaxation processes, adverse to SMM properties, can be effectively suppressed by high-symmetry crystal fields (*D*_4d_, *D*_5h_, and *D*_6h_ coordination geometries) [25,26], and the rigidity of equatorial-plane ligands that hinders molecular vibration and inhibits Raman relaxation. For instance, a stable *D*_6h_ dysprosium single-molecule magnet (Dy-SMM) incorporating a rigid hexaazamacrocyclic ligand exhibits an effective energy barrier (*U*_eff_) as high as 1833 K and an open hysteresis loop at 20 K [27]. In contrast, well-known high-temperature Dy-SMMs [Dy(Cp*)_2_]^+^ (Cp* = {C5Me5}^−^) with an open hysteresis loop of up to 80 K are not stable under aerobic conditions and are hard to synthesize [12].

**Scheme 1:**
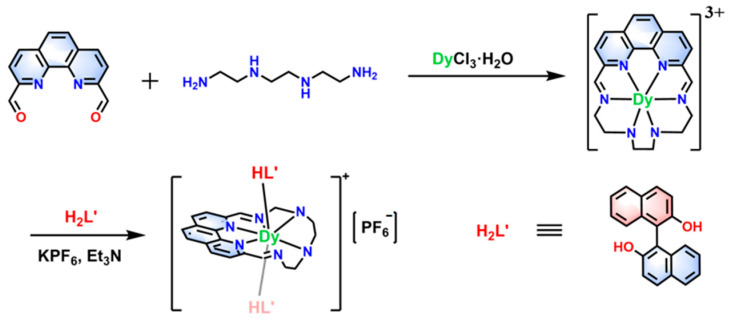
Synthetic route for **1**.

Due to this interest in multi-functional and stable *D*_6h_ Dy-SMMs, we made an effort to prepare such species by incorporating chiral and fluorescent ligands. With the help of theoretical calculations, we can deeply understand the magneto-structural correlation of *D*_6h_ Dy-SMMs. In this work, we report a mononuclear octa-coordinate complex [Dy(phenN_6_)(HL′)_2_]PF_6_·CH_2_Cl_2_ (H_2_L′ = *R*/*S*-1,1′-binaphthyl-2,2′-diphenol), featuring local *D*_6h_ symmetry. The flexible cyclic polydentate ligand phenN_6_ (Scheme 1) provides a weak coordination environment in the equatorial plane, forming a non-planar coordination sphere around Dy^3+^. The axial positions are occupied by chiral fluorescent phenoxy ligands (HL′)^−^, generating two short Dy-O_phenoxy_ coordination bonds. By constructing a pseudo-*D*_6h_ local configuration with strong axial coordination bonds, the magnetic anisotropy along the axial direction is maximized, thereby enhancing the effective energy barrier (*U*_eff_). AC susceptibility measurements reveal distinct temperature-dependent and frequency-dependent characteristics in the AC magnetic signals, typical of single-molecule magnet behavior.

## 2. Results and Discussion

### 2.1. Synthesis and Circular Dichroism Spectra

The electrostatic repulsion model suggests that the strategy most ideal for enhancing the magnetic performance of flattened Dy^3+^ ions involves concentrating ligands in the axial direction to form a monodentate coordination compound (coordination number = 1). However, lanthanide ions exhibit diverse coordination numbers and complicated configurations, making it challenging to obtain even bidentate structures, let alone monodentate ones. In order to obtain a *D*_6h_ high-performance Dy(III) SMM, we adopted a two-step strategy. This involved intentionally weakening equatorial coordinating atoms using a hexaazamacryclic ligand (phenN_6_) and then strategically enhancing the axial coordination by positioning the phenoxyl oxygen atoms of HL^−^ along the anisotropy axis [17,21]. The two-step synthesis of complex **1** is shown in Scheme 1. Through the reaction of HL^−^ with Dy(phenN_6_)Cl_3_ in CH_2_Cl_2_, we successfully obtained complex **1**, which exhibits local *D*_6h_ symmetry. The purity of the complex was confirmed by micro-elemental CHN analyses. The crystals show the partial loss of the CH_2_Cl_2_ solvent and slight solubility in acetonitrile. The thermogravimetric analysis (TGA) of complexes **1**(***R***/***S***) reveals that all CH_2_Cl_2_ solvents are gradually lost from room temperature to 185 °C (Figure S1). The infrared spectrum of complex **1** shows strong absorption peaks at 1600 cm^−1^ and 830 cm^−1^, which are due to the C=N stretching vibration of the Schiff base ligand and the P-F stretching vibration in PF_6_^−^ ions (Figure S2). The fluorescence spectra of complexes **1**(***R***) and **1**(***S***) in the acetonitrile solution (1 × 10^−5^ M) with an excitation wavelength of 320 nm show the characteristic emission at 360 nm for 1,1′-bi-2-naphthol (Figure S3).

**Figure 1:**
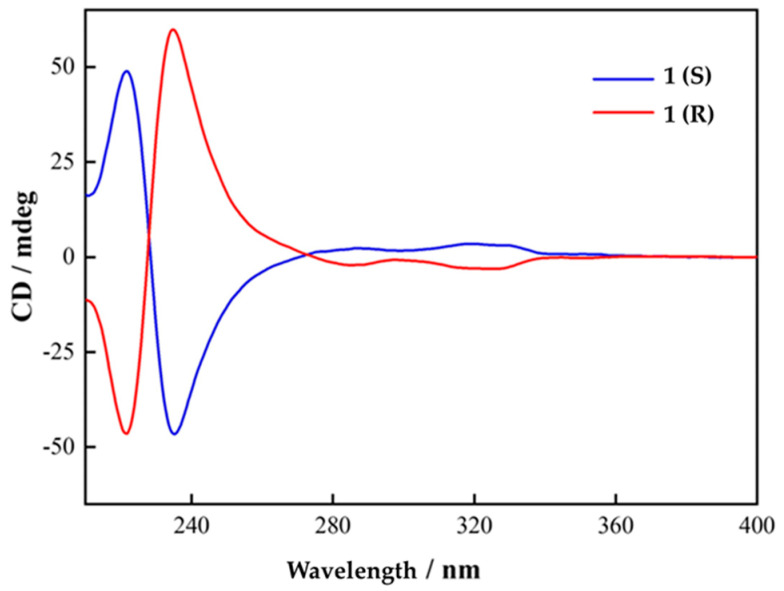
The CD diagram of complex **1** in acetonitrile ([**1**] = 5 × 10^−6^ M).

The chirality of ligands can produce chiral complexes through coordination. 1,1′-bi-2-naphthol is a common chiral compound. After coordination with Dy^3+^, a pair of chiral mononuclear complexes, **1**(***R***) and **1**(***S***), are formed, which are mirror images of each other in their crystal structures. The circular dichroism (CD) spectra in acetonitrile solution further prove that they are enantiomers. As shown in Figure 1, the CD spectrum of the complex **1**(***R***) shows a positive Cotton effect at 221 nm and a negative Cotton effect at 235 nm, which arises from the π → π* charge transfer in the binaphthyl benzene ring. Conversely, the CD spectrum of complex **1**(***S***) shows a mirror-image signal completely opposite to that of **1**(***R***) at the same wavelength, indicating that **1**(***R***) and **1**(***S***) are a pair of Dy(III) enantiomers.

### 2.2. Structure

**Figure 2:**
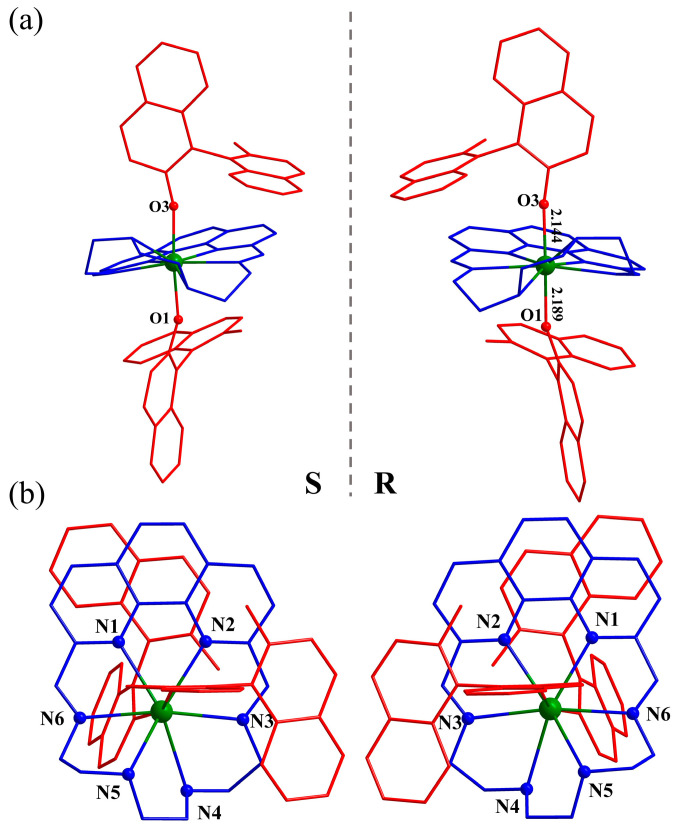
The crystal structure diagrams of complexes **1**(***S***) and **1**(***R***): (**a**) side view; (**b**) top view. The lattice solvents and hydrogen atoms are omitted for clarity.

**Figure 3:**
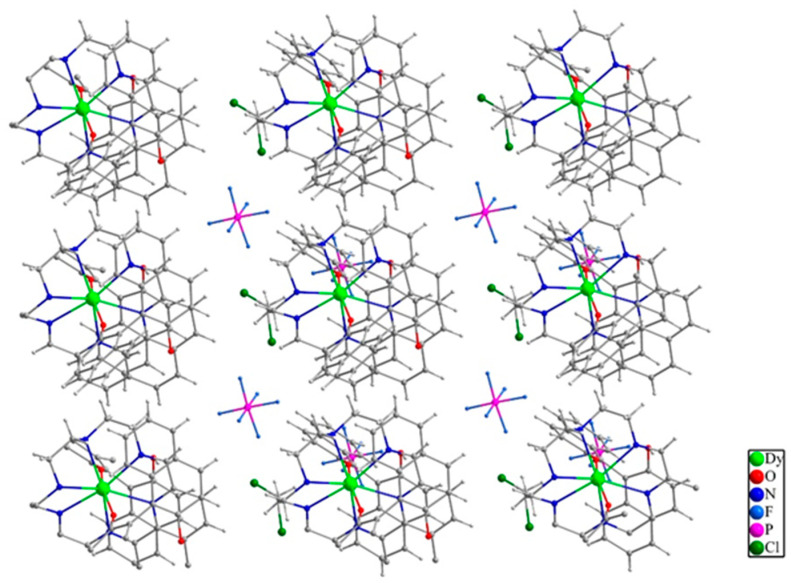
The molecular stacking diagram of complex **1**(***R***).

Mononuclear complexes **1**(***R***) and **1**(***S***) crystallize in polar space group *P*1, and the crystallographic data are summarized in Table S1. Selected bond distances and bond angles are given in Table S2. The molecular structure of the [Dy(phenN_6_)(HL)_2_]^+^ cation for complexes **1**(***R***) and **1**(***S***) is depicted in Figure 2. The chirality of the complexes, corresponding to *S*/*R* configurations, can be determined by the orientation of the uncoordinated phenol oxygen bonds in the 1,1′-binaphthyl-2,2′-diphenol ligands. The independent asymmetric unit contains a Dy^3+^ ion, a neutral cyclic ligand (phenN_6_), two monovalent axial ligands (HL′)^−^, a charge-balancing anion PF_6_^−^, and a lattice solvent molecule (CH_2_Cl_2_). As designed, Dy^3+^ is indeed encapsulated within the coordination cavity formed by the hexaaza N_6_ ligand. The rigid *o*-phenanthroline constrains the equatorial coordination atoms to be coplanar at the head of the macrocycle, while the flexible aliphatic amine chain at the tail deviates from the equatorial plane upon coordination (N_5_), resulting in a non-planar equatorial coordination. A similar distortion can be observed in less distorted complexes [Dy(bpyN_6_)(Ph_3_SiO)_2_](BPh_4_) and [Dy(phenN_6_)(Ph_3_SiO)_2_](PF_6_) [17,21]. The axial positions are occupied by the phenoxy group of binaphthol ligands. According to the SHAPE calculation (version 2.1), the local geometry of Dy^3+^ is close to the compressed hexagonal bipyramidal *D*_6h_ configuration with a deviation parameter of 5.478 (Table S3). The axial Dy-O_phenoxy_ bond distances are 2.189(5) and 2.144(5) Å, respectively, while the bond lengths of Dy-N in the equatorial plane are in the range of 2.524(7)–2.717(5) Å, which are significantly longer than the axial Dy-O_phenoxy_ bond lengths for **1**(***R***). The axial O_phenoxy_-Dy-O_phenoxy_ bond angle of 162.91(17)° for **1**(***R***) deviates markedly from the ideal linearity, likely due to the spatial hindrance induced by the non-planarity of the equatorial phenN_6_ ligand. In the crystal lattice, free CH_2_Cl_2_ and PF_6_^−^ are situated adjacent to the [Dy(phenN_6_)(HL)_2_]^+^ cations (Figure 3) and form abundant weak intermolecular interactions. The nearest intermolecular Dy---Dy distance is 9.224 Å for **1**(***R***).

### 2.3. Magnetism

**Figure 4:**
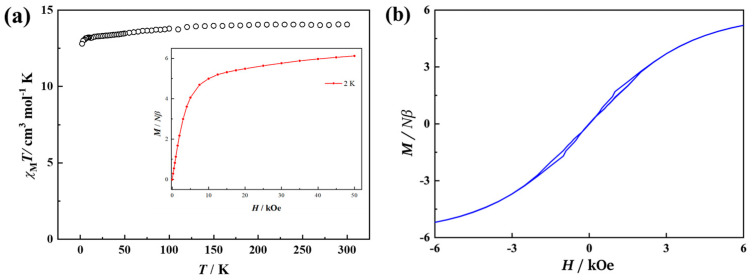
(**a**) The temperature-dependent magnetic susceptibilities (*χ*_M_*T*) under a DC field of 1000 Oe. Inset: field dependences of magnetization at 2 K; (**b**) hysteresis loop at 1.9 K.

Variable-temperature magnetic susceptibility measurements for complex **1** were conducted under a 1000 Oe DC field across the 2–300 K range. As shown in Figure 4a, the room-temperature *χ*_M_*T* value of 13.9 cm^3^·mol^−1^·K is close to the theoretical value (14.17 cm^3^·mol^−1^·K) for a non-interacting Dy^3+^ ion (^6^*H*_15/2_, *S* = 5/2, *L* = 5, *g*_J_ = 4/3). The *χ*_M_*T* profile of mononuclear complex **1** remains nearly constant upon cooling before gradually decreasing to a minimum of 11.79 cm^3^·mol^−1^·K at 2 K, which is indicative of weak intermolecular antiferromagnetic interactions between Dy^3+^ centers. The magnetization curve of complex **1** at 2.0 K (Inset of Figure 4a) shows that the magnetization in the region of 0–10 kOe increases nearly linearly with the increase in the external magnetic field, and then gradually to 5.6 *Nβ* at 50 kOe, which is far lower than the theoretical saturation value of 10 *Nβ* (*g*_J_ × J) for Dy(III) complexes. As shown in Figure 4b, complex **1** has a small hysteresis loop at 1.9 K without residual magnetization at the external zero dc field.

**Figure 5:**
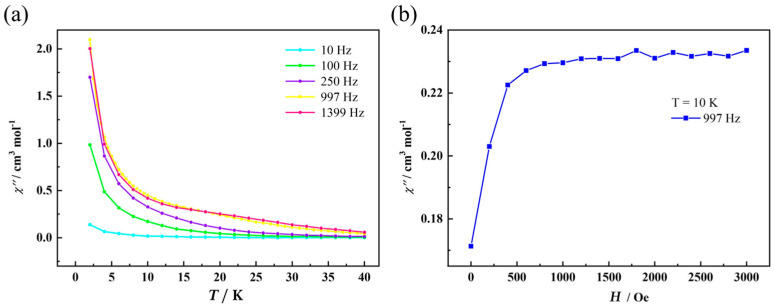
(**a**) Temperature-dependent out-of-phase alternating current (ac) magnetic susceptibilities of complex **1** under a zero applied DC field; (**b**) field-dependent *χ*″ for complex **1** at 10 K and 997 Hz.

The magnetic susceptibilities (*χ*″) of the temperature-dependent out-of-phase alternating current (ac) of complex **1** under the zero DC field are shown in Figure 5a. The presence of non-zero *χ*″ signals across the 10–997 Hz range, in the absence of distinct peaks, suggests that significant quantum tunneling of magnetization (QTM) exists. To mitigate this effect, field-dependent AC susceptibility measurements were conducted at 10 K and 997 Hz to identify the optimal suppressing DC field. As revealed in Figure 5b, the *χ*″ response exhibits a maximum value near 1800 Oe, indicating effective QTM suppression in this field. Subsequent AC susceptibility tests were thus performed under an applied 1800-Oe DC field.

**Figure 6:**
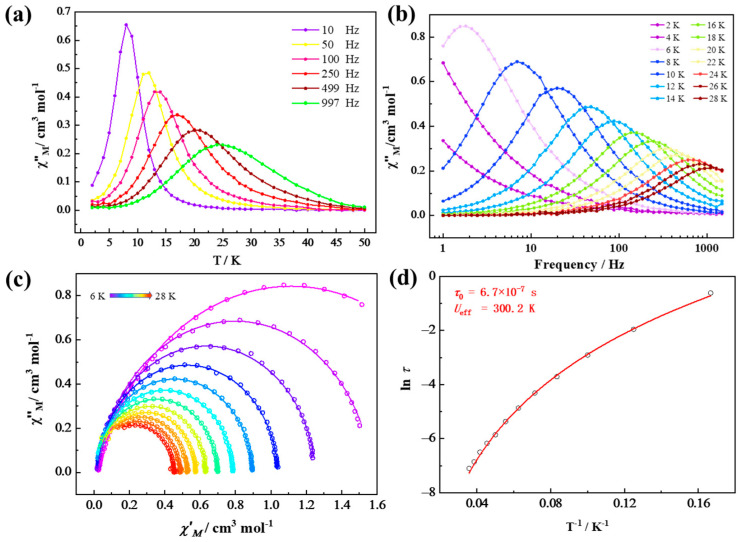
The dynamic magnetic behavior of complex **1** under the applied DC field of 1800 Oe: (**a**) the temperature-dependent imaginary AC susceptibility *χ*_M_″; (**b**) frequency-dependent *χ*_M_″ curves; (**c**) Cole–Cole plots, where the solid lines are the fitting results; and (**d**) the lnτ vs. *T*^−1^ plot, where the solid line is the fitting result.

Figure 6 demonstrates the temperature- and frequency-dependent out-of-phase (*χ*_M_″) AC susceptibility signals for **1**, which are characteristic of single-molecule magnet (SMM) behavior. Cole–Cole plots between 6.0 and 28.0 K (Figure 6c) display semicircular profiles that were well fitted using the generalized Debye model (Table S4). The small α values (α < 0.17) manifest a narrow distribution of relaxation time.

An Arrhenius analysis of the extracted relaxation times (ln*τ* vs. *T*^−1^) reveals two distinct regimes: (i) a linear high-temperature region (*T* > 15 K) governed by the Orbach process, and (ii) a curved low-temperature region (*T* < 15 K) dominated by a Raman relaxation mechanism (Figure 6d). Consequently, the entire temperature range was fitted using Equation $\tau^{-1}=CT^{n}+\tau_{0}^{-1}\exp\left(-\frac{U_{eff}}{k_{B}T}\right)$ incorporating both Orbach and Raman relaxation mechanisms, yielding the parameters of *n* = 4.24(12), *C* = 0.001(1) s^−1^·K^−*n*^, *U*_eff_ = 300.2(20) K, and *τ*_0_ = 6.7(3) × 10^−7^ s. For classical Raman relaxation, *n* varies in the range of 2–9 for a phonon bottleneck (*n* = 2) and an ideal Kramers ion (*n* = 9). In many SMMs, *n* may be smaller when acoustic and optical phonons are present (usually *n* = 2–6, Table S5) [17,19,20,28].

In order to investigate the magneto-structural correlation, *D*_6h_ Dy-SMMs (37 cases) with *U*_eff_ values in the range of 35–2437 K were collected (Table S5) [17,18,19,20,21,27,29,30,31,32,33,34,35,36,37,38,39]. It is well documented that for mononuclear *D*_6h_-Dy single-molecule magnets, the rigidity and electrical properties of the equatorial ligands, the electronegativity of the coordination atoms, and the steric hindrance of the axial ligands change the structure and local coordination configuration (coplanarity) of the complex, and then alter the strength of the crystal field around Dy(III), thus regulating the magnetic anisotropy of Dy(III). A comparison of the magnetic properties for *D*_6h_ high-performance Dy-SMMs in Table S5 suggests that the axial O-Dy-O bond angle plays the most critical role, i.e., larger bond angles correspond to better magnetic anisotropy and higher *U*_eff_. In addition, the coplanarity, electrical neutrality, and electronegativity of equatorial coordination atoms play a favorable secondary role. In complex **1**, the Dy^3+^ center adopts a compressed *D*_6h_ configuration with a neutral hexaaza macrocyclic ligand, showing a medium *U*_eff_ among similar pseudo-*D*_6h_ Dy-SMMs (Table S5). The small axial O-Dy-O bond angle of 162.89(18)° for **1** should be responsible for the situation. Previous theoretical calculation results indicate that the large deviation from the ideal *D*_6h_ usually brings about the QTM process occurring in the first excited state, which leads to the failure of the energy barrier flip in the second excited state and, finally, a small effective energy barrier. In contrast, an ideal *D*_6h_ could make the anisotropic axis of the first excited state and even the higher excited states coincide with the ground state, and the QTM in the first or higher excited states can be effectively suppressed, thus significantly improving the effective energy barrier [34].

**Figure 7:**
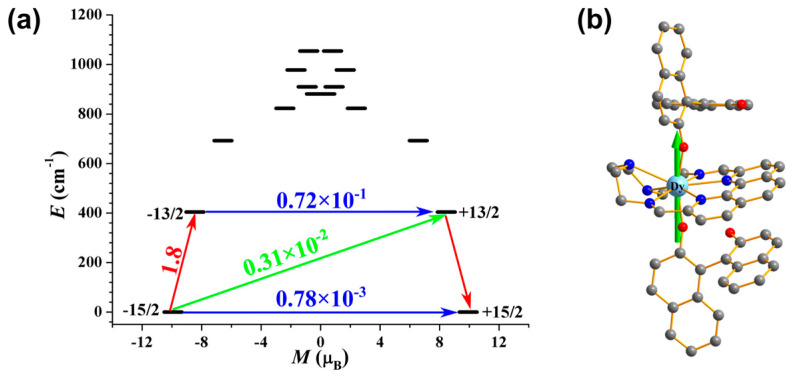
(**a**) The magnetization blocking barrier of complex **1** with the KDs (thick black lines) as a function of their magnetic moment along the magnetic axis. The blue lines correspond to the diagonal matrix element of the transversal magnetic moment; the green lines represent Orbach relaxation processes. The red arrows represent the most probable path for magnetic relaxation in the corresponding compounds. The numbers at each arrow stand for the mean absolute value of the corresponding matrix element of the transition magnetic moment. (**b**) The calculated orientation of the local main magnetic axis on Dy^III^ ion of complex **1** in its ground KD.

Complete-active-space self-consistent field (CASSCF) calculations on complex **1** (Figure S4) on the basis of an X-ray-determined geometry were carried out with OpenMolcas [40] and SINGLE_ANISO [41,42,43] programs (see Supporting Information for details) to deeply understand the relaxation mechanism of complex **1**. The energy levels (cm^−1^), *g* (*g*_x_, *g*_y_, *g*_z_) tensors, and predominate *m*_J_ of the lowest eight Kramers doublets (KDs) of complex **1** are shown in Table S6, where *m*_J_ is equal to ±15/2 in its ground Kramers doublets (KD_0_) with *g*_z_ ≈ 20.000 > *g*_x,y_ ≈ 0.000, indicating a nearly perfectly axial anisotropy for **1**. The mixed *m*_J_ components for the lowest eight KDs of **1** (Table S7) show that the KD_0_ is mostly composed of *m*_J_ = ±15/2, leading to a small transversal magnetic moment in the KD_0_. However, the first excited state KD_1_ for **1** comprises 75.9%|+13/2> and 22.6%|−13/2>, which causes a large transversal magnetic moment within KD_1_, as shown in Figure 7a. As expected, the main magnetic axis on the Dy^III^ ion of **1** in the KD_0_ is aligned along the O-Dy-O direction in Figure 7b.

In Figure 7a, the transversal magnetic moment in the KD_0_ for **1** is 0.78 × 10^−3^ *µ*_B_, which is too small, meaning a fast QTM in the KD_0_ is suppressed at low temperatures. By contrast, the transversal magnetic moment in the KD_1_ is 0.72 × 10^−1^ *µ*_B_, allowing for a fast thermal-assisted QTM. Hence, the magnetic relaxation of **1** can probably proceed through KD_1_. Thus, the calculated magnetization blocking barrier for **1** is 404.1 cm^−1^, which is higher than the experimental energy barrier value of 300.2 K (208.6 cm^−1^). The difference is due to unfavorable effects, such as anharmonic phonons, Raman magnetic relaxation, QTM, etc., on the energy barrier, which has been frequently observed in Dy-SMMs. Nevertheless, the calculation results for **1** suggest that the short axial Dy-O_phenoxy_ distances of 2.189(5) and 2.145(5) Å give rise to large energy splitting between KD_1_ and KD_0_, while the distortion from perfect *D*_6h_ leads to relaxation through KD_1_. Obviously, this phenomenon is consistent with the experimental results.

## 3. Materials and Methods

### 3.1. Synthesis

All of the reagents were commercially available and were used without further purification.

#### 3.1.1. Synthesis of the Precursor phenN_6_-DyCl_3_

In total, 1.18 g (5.00 mmol) of 1,10-*o*-phenanthroline-2,9-dicarbaldehyde was dissolved in 30 mL of hot ethanol; then, 800 μL (5.00 mmol) of triethylenetetramine was added, and the reaction system began to slowly produce turbidity. Then, 1.92 g (5.00 mmol) of dysprosium chloride hexahydrate was added. The mixture was heated to reflux for 6 h. After the reaction, a large amount of light brown precipitate was produced at the bottom of the round-bottomed flask. The reaction product was collected by suction filtration and was repeatedly washed with ice ethanol. Finally, the potential coordination solvent was removed by vacuum drying at 80 °C for 5 h, and finally, about 1.80 g (2.9 mmol) of brown powder phenN_6_-DyCl_3_ was obtained with a yield of about 65%.

#### 3.1.2. Synthesis of Complexes [Dy(phenN_6_)(HL′)_2_]PF_6_·CH_2_Cl_2_ (**1*R***/**1*S***)

The precursors phenN_6_-DyCl_3_ (30.8 mg, 0.0500 mmol), *R*/*S*-1,1′-binaphthyl-2,2′-diphenol (H_2_L′, 28.6 mg, 0.100 mmol), KPF_6_ (18.4 mg, 0.100 mmol), and triethylamine (13.8 μL, 0.100 mmol) were, respectively, added into 10 mL of dichloromethane solvent, and then 10 mL of deionized water was added into the mixed system under stirring at room temperature. Subsequently, the reaction system was refluxed for 2 h. The solution was allowed to cool at room temperature. The dichloromethane layer was separated and filtered, and the crimson filtrate was allowed to stand and evaporate slowly in a small bottle with a hole. Two days later, red, flaky crystals (34.7 mg, 0.0265 mmol) were precipitated with a yield of about 53% (based on the amount of precursor phenN_6_-DyCl_3_). Elemental analysis (%) was calcd for C_61_H_50_Cl_2_DyF_6_N_6_O_4_P (FW = 1309.44 g·mol^−1^): C, 55.95; H, 3.85; and N, 6.42. The following were measured: C, 55.8; H, 3.7; and N, 6.8. IR (KBr disks, cm^−1^): 1600 (s), 830 (s). TGA yielded the following: 4.5% (-CH_2_Cl_2_, 4.5% calcd).

### 3.2. Physical Measurements

Single-crystal X-ray data were collected by Rigaku SuperNova, Dual, Cu at zero, and AtlasS2, Rigaku Holdings Corporation, Tokyo, Japan. We used the Olex2 program (version 1.3) to solve the structure and used the full matrix least square method based on F^2^ to refine it with the method of SHEXL-2018/3. Hydrogen atoms were added geometrically and refined by the riding model. The temperature- and field-dependent magnetic susceptibility were measured by the MPMS XL5 SQUID magnetometer of Quantum Design Company, San Diego, CA, USA. Infrared spectra (KBr tablet) in the range of 400~4000 cm^−1^ were recorded on WQF 510A FTIR equipment (EnviSense, Lublin, Poland), and the scanning interval was 2 cm^−1^. Thermogravimetric analyses were performed on a METTLER TOLEDO TGA/DSC1 instrument (METTLER TOLEDO, Hong Kong, China) in the temperature range of 30–800 °C using a heating rate of 10 K·min^−1^ under N_2_ atmosphere. The photoluminescence spectrum was measured by a Lengguang F98 fluorescence spectrophotometer (Shanghai, China). The scanning speed was 1000 nm/min, and the scanning interval was 1 nm. The circular dichroism (CD) spectrum was measured by the JASCO J-1500 spectrometer of JASCO Corporation, Tokyo, Japan.

## 4. Conclusions

A chiral mononuclear complex [Dy(phenN_6_)(HL′)_2_]PF_6_·CH_2_Cl_2_ (H_2_L′ = *R*/*S*-1,1′-binaphthyl-2,2′-diphenol) with local *D*_6h_ symmetry was successfully constructed. The short Dy–O_phenoxy_ coordination bonds formed through the axial coordination of chiral HL’^−^ ligands effectively enhanced magnetic anisotropy. AC magnetic susceptibility measurements unambiguously confirmed the single-molecule magnet behavior with an effective energy barrier of 300.2 K under 1800 Oe. This work reemphasizes the fact that the perfect *D*_6h_ coordination configuration of Dy(III) is in favor of high-performance Dy-SMMs. Future work will involve the preparation of new multi-functional Ln-SMMs with sensitized rare earth luminescence and improved SMM performance.

## Data Availability

The original contributions presented in this study are included in the article/Supplementary Materials. Further inquiries can be directed to the corresponding author (H.-Z.K.).

## Supplementary Materials

The following supporting information can be downloaded at https://www.mdpi.com/article/10.3390/molecules30092043/s1. Figure S1: The thermogravimetric curves of complex 1(*R*/*S*); Figure S2: Infrared spectrum of complex 1(*R*/*S*); Figure S3: Fluorescence emission spectra of complex **1** in acetonitrile (1 × 10^−5^ M); Figure S4: Molecular structure of the cation for complex **1** used for CASSCF calculations; H atoms are omitted for clarify; Table S1: Crystallographic data of complexes **1*R***/***S***; Table S2: Selected bond distances (Å) and bond angles (°) in complexes **1**(***R***) and **1**(***S***); Table S3: Coordination geometry calculated by SHAPE 2.1 for complex **1*R***; Table S4: Cole–Cole fitting parameters under the 1800 Oe DC field for complex **1**; Table S5: Structural information and the effective energy barrier for *D*_6h_ Dy(III) SMMs; Table S6. Calculated energy levels (cm^−1^), *g* (*g*_x_, *g*_y_, *g*_z_) tensors, and the predominate *m*_J_ of the lowest eight Kramers doublets (KDs) of complex **1** using CASSCF/RASSI-SO with OpenMolcas; Table S7. Wave functions with the definite projection of the total moment |*m*_J_ > for the lowest eight KDs of complex **1** using CASSCF/RASSI-SO with OpenMolcas. References [44,45] are cited in the Supplementary Materials.
