# Supplementary material for: Chiral Pseudo-D6h Dy(III) Single-Molecule Magnet Based on a Hexaaza Macrocycle
Source: Molecules. 2025 May 3;30(9):2043. doi: 10.3390/molecules30092043 (PMC12073781; doi:10.3390/molecules30092043)
Supplement: Supplementary file 1 [file molecules-30-02043-s001.zip › molecules-3595564-supplementary.pdf]

Supporting Information

Chiral Pseudo-D_{6h} Dy(III) Single-Molecule Magnet Based on a Hexaaza Macrocycle

Jia-Hui Liu, Yi-Shu Jin, Jinkui Tang,* Cai-Ming Liu, Yi-Quan Zhang,* Hui-Zhong Kou*

1. Engineering Research Center of Advanced Rare Earth Materials (Ministry of Education), Department of Chemistry, Tsinghua University, Beijing 100084, China
2. State Key Laboratory of Rare Earth Resource Utilization Changchun Institute of Applied Chemistry, Chinese Academy of Sciences, Renmin Street 5625, Changchun 130022, China
3. Beijing National Laboratory for Molecular Sciences, Center for Molecular Science, Institute of Chemistry, Chinese Academy of Sciences, Beijing 100190, China; cmliu@iccas.ac.cn
4. Ministry of Education Key Laboratory of NSLSCS, School of Physical Science and Technology, Nanjing Normal University, Nanjing 210023, China.

* Correspondence: tang@ciac.ac.cn, zhangyiquan@njnu.edu.cn, kouhz@mail.tsinghua.edu.cn

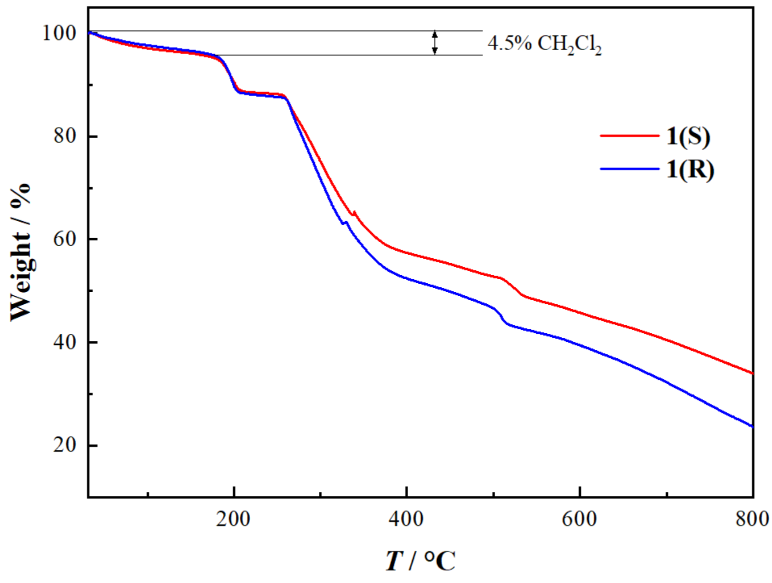

Figure S1. The thermogravimetric curves of complex **1(R/S)**.

We tested the complexes **1(R)** and **1(S)** by TGA in order to explore their thermal stability. As can be seen from Figure S1, the sample slowly loses the lattice solvent CH_2Cl_2 from room temperature, and above 185 °C solvent completely leaves (weight loss is 4.5 %). Above 185°C, the complex begins to decompose.

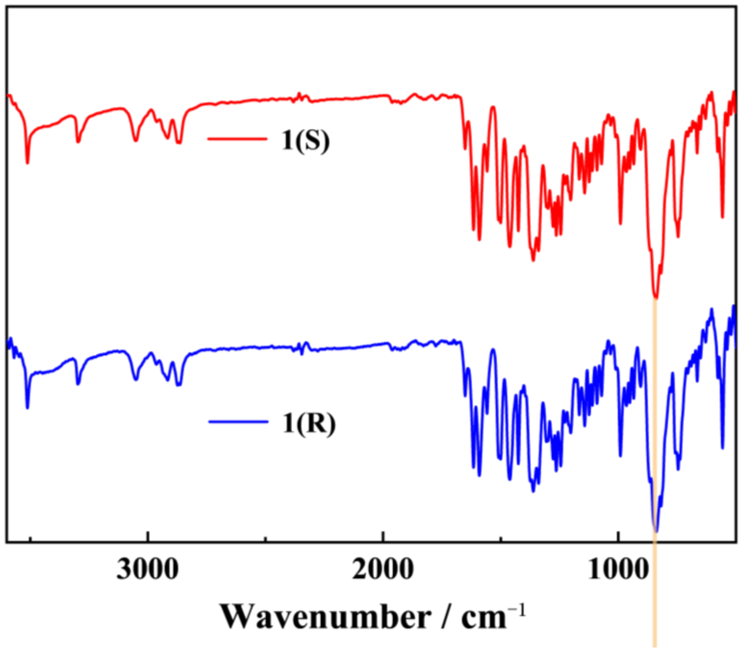

Figure S2. Infrared spectrum of complex **1(R/S)**.

As shown in Figure S2, the infrared spectrum of complex **1** shows a strong absorption peak at 1600 cm^{-1}, which may be caused by the stretching vibration of Schiff base ligand C=N, while a strong absorption peak at 830 cm^{-1} is attributed to the P-F stretching vibration in PF_6^- ion.

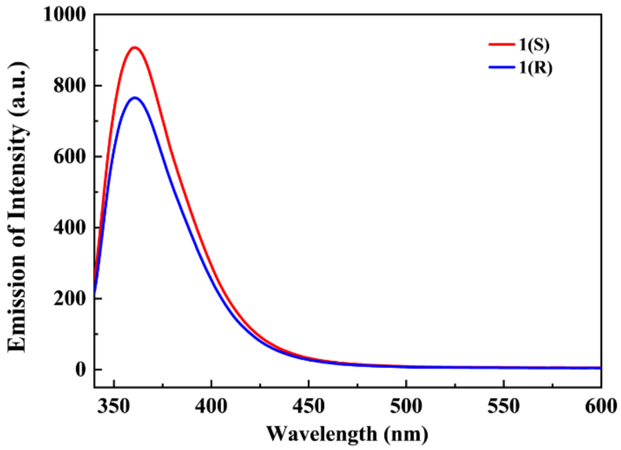

Figure S3. Fluorescence emission spectra of complexes **1** in acetonitrile (1×10^{-5} M)

The fluorescence spectra of complexes **1(R)** and **1(S)** in acetonitrile solution with excitation wavelength of 320 nm are shown in Fig. S3. It can be seen that they all have tiny shoulder peaks around 400 nm, which are derived from the rigid aromatic ring of equatorial plane ligands. 1,1'-binaphthyl-2,2'-diphenol is a good fluorescent chromophore, and the dihedral angle of its monomer is close to 90°, which can effectively prevent intermolecular π-π stacking, thus obtaining strong fluorescence emission. Complex **1(R/S)** has the maximum emission peak at 360 nm, which is the characteristic emission peak of naphthalene ring itself.

Table S1. Crystallographic Data for Complexes **1R/S**.

	**1**R	**1**S
Formula	$C_{61}H_{50}Cl_2DyF_6N_6O_4P$	$C_{61}H_{50}Cl_2DyF_6N_6O_4P$
Formula weight	1309.44	1309.44
Temperature (K)	109.1(5)	173.0(1)
Crystal system	triclinic	triclinic
Space group	$P1$	$P1$
a (Å)	9.2245(4)	9.2486(5)
b (Å)	12.0444(5)	12.0803(5)
c (Å)	12.7704(5)	12.8072(5)
α (°)	72.842(4)	72.819(4)
β (°)	77.784(3)	77.863(4)
γ (°)	88.117(3)	88.014(4)
V (Å^3)	1324.33(10)	1335.91(11)
Z	1	1
ρ_{calc} (g/cm^3)	1.642	1.628
R_{int}	0.0380	0.0316
F(000)	659	659
Radiation	CuKα (λ = 1.54178)	CuKα (λ = 1.54184)
Flack	-0.004(3)	-0.002(4)
GOF on F^2	1.036	1.053
R_1	0.0362	0.0418
wR_2 (all data)	0.0900	0.1069
CCDC	2425772	2425773

Table S2. Selected Bond Distances (Å) and Bond Angles (°) in Complexes **1(R)** and **1(S)**.

	**1(R)**	**1(S)**
Dy1-O1	2.189(5)	2.214(7)
Dy1-O3	2.144(5)	2.127(7)
Dy1-N1	2.531(6)	2.533(7)
Dy1-N2	2.543(6)	2.560(7)
Dy1-N3	2.555(7)	2.543(7)
Dy1-N4	2.604(6)	2.608(7)
Dy1-N5	2.717(5)	2.723(7)
Dy1-N6	2.524(7)	2.523(8)
O1-Dy1-O3	162.91(17)	163.3(2)

Table S3. Coordination Geometry Calculated by SHAPE 2.1 for Complex **1R**.

Configuration	ABOXIY
Octagon (D_{8h})	32.308
Heptagonal pyramid (C_{7v})	21.307
Hexagonal bipyramid (D_{6h})	5.478
Cube (O_h)	7.711
Square antiprism (D_{4d})	8.074
Triangular dodecahedron (D_{2d})	6.437
Johnson gyrobifastigium J26 (D_{2d})	5.650
Johnson elongated triangular bipyramid J14 (D_{3h})	23.826
Biaugmented trigonal prism J50 (C_{2v})	7.088
Biaugmented trigonal prism (C_{2v})	6.978
Snub diphenoid J84 (D_{2d})	7.570
Triakis tetrahedron (T_d)	8.393
Elongated trigonal bipyramid (D_{3h})	23.826

Table S4. Cole-Cole Fitting Parameters Under 1800 Oe dc Field for Complex **1**.

T (K)	τ (s)	α	χ_t ($cm^3 \cdot mol^{-1}$)	χ_s ($cm^3 \cdot mol^{-1}$)
6.0	0.58151	0.16177	2.20475	0.0247
8.0	0.13478	0.07685	1.57055	0.02447
10.0	0.04836	0.04671	1.25194	0.0205
12.0	0.02144	0.03267	1.03954	0.01786
14.0	0.01139	0.02567	0.89577	0.01569
16.0	0.00667	0.02379	0.78542	0.01336
18.0	0.00425	0.02324	0.69959	0.012
20.0	0.00286	0.02642	0.63129	0.00719
22.0	0.00208	0.01684	0.57458	0.01682
24.0	0.0015	0.02632	0.52702	0.00371
26.0	0.00115	0.02753	0.48786	0.00745
28.0	0.00090	0.02717	0.45371	0.00756

Table S5. Structural Information and the Effective Energy Barrier for D_{6h} Dy(III) SMMs

Complex	Axial Dy-O/Å	Axial O-Dy-O/Cl/°	U_{eff}/K	H_{dc}/Oe	n	Functionality	Reference[a]
$[Dy(18\text{-}C\text{-}6)(O^tBu)_2][I_3]$	2.068(5)	177.5(1), 177.5(2)	2352	0	3.9	no	31
$[Dy(18\text{-}C\text{-}6)(1\text{-}AdO)_2][I_3]$	2.054(6)	177.8(2), 177.5(2)	2427	0	4.1	no	31
RRRR-Dy-$D_{6h}F_{12}$	2.124(6), 2.139(7)	178.3(3)	1833	0	3.2	no	27
SSSS-Dy-$D_{6h}F_{12}$	2.122(6), 2.147(6)	177.9(3)	1819	0	3.0	no	27
$[Dy(L1^{N6})(Ph_3SiO)_2][OTf]$	2.129(4), 2.142(4)	177.05(15)	1680	0	2.00	no	18
$[Dy(L1^{N6})(Ph_3SiO)_2]ClO_4$	2.138(2), 2.141(2)	175.53(8)	1732	0	2.08	no	18
$[Dy(L2^{N6})(Ph_3SiO)_2](3\text{-}BrBPh_4)$	2.131(4), 2.138(4)<br>2.133(5), 2.137(5)	175.4(2)<br>175.0(3)°	1363<br>1415	0	2.03<br>2.00	chiral	18
$[Dy(L2^{N6})(Ph_3SiO)_2](4\text{-}BrBPh_4)$	2.136(7), 2.145(7)<br>2.140(6), 2.141(5)	178.4(4)<br>179.1(3)	1369<br>1434	0	2.05<br>2.00	chiral	18
$[Dy(L^E)(4\text{-}MeO\text{-}PhO)_2]BPh_4$	2.089(6)	180	1338	0	2.82	no	30
$[Dy(phenN4O2)(Ph_3SiO)_2](PF_6)$	2.142(2), 2.153(2)	174.52(7)	1360	0	2.45	no	21
$[Dy(L^{N3O3})(Ph_3SiO)_2](PF_6)$	2.147(2), 2.163(2)	175.84(8)	1300	0	2.12	no	39
$[DyL^{1NH}(Ph_3SiO)_2]BPh_4$	2.158(14),<br>2.169(15)	173.7(7)	1270	0	2.2	no	19
$[DyL^{2O}(Ph_3SiO)_2]BPh_4$	2.141(5), 2.130(5)	172.4(2)	1229	0	2.3	no	19
$[LnNi_2(L)_2(saldt)_2(py)_2](ClO_4)$	2.114(3), 2.127(3)	172.16(11)	1237	0	3.24	no	37
$[Dy(L^E)(naPhO)_2]BPh_4$	2.084(6), 2.100(6)	168.9(2)	1226	0	3.2	no	30
$[Dy(L^E)(PhO)_2]BPh_4$	2.108(6)	180	1100	0	2.66	no	30
$[DyL^{1N6}(Ph_3SiO)_2](BPh_4)$	2.16(1), 2.15(1)	175.10(18)	1092	0	3.98	no	38
$[Dy(18\text{-}C\text{-}6)Cl(O^tBu)]BPh_4$	2.075(8)/2.043(4)	174.9(6)/165.7(2)	1000	0	2.8	no	36
$[DyL^{N6}{}_R(L2)_2]BPh_4$	2.123(3), 2.139(3)	178.06(16)	1011	0	2.51	chiral	34
$[Dy(L^{N6})(Ph_3SiO)_2](PF_6)$	2.153(7), 2.163(6)	179.8(2)	1080	0	2.32	no	29
$[Dy(L^{N6})(Ph_3SiO)_2](BPh_4)$	2.142(2), 2.151(2)	176.13(6)	1124	0	2.95	no	29
$[Dy(L^{N6})(2,4\text{-}di\text{-}tBu\text{-}PhO)_2](PF_6)$	2.130(1), 2.146(1)	176.54(5)	973	0	2.50	no	29
$[DyL^{3NMe}(Ph_3SiO)_2]BPh_4$	2.125(7), 2.160(8)	165.2(3)	989	0	2.0	no	19
$[DyL^{4FN6}(Ph_3SiO)_2](BPh_4)$	2.124(3), 2.150(3)	175.13(12)	946	0	4.26	no	38
$[Dy(L^{N6})(Ph_3SiO)_2](PF_6)$	2.143(7), 2.152(7)	168.6(3)	989	0	2.57	no	39
$[Dy(phenN6)(Ph_3SiO)_2](PF_6)$	2.159(5), 2.163(5)	164.6(2)	779	0	2.42	no	21
$[Dy(bpyN4)(Ph_3SiO)_2](BPh_4)$	2.139(4), 2.143(4)	169.06(17)	584	0	2.46	no	17
$[Dy(L^1{}_R)(4\text{-}Me\text{-}PhO)_2]BPh_4$	2.136(4), 2.145(4)	170.45(17)	556	0	1.75	chiral	32
$[Dy_3L^x{}_2(phenol)_5]$	2.055(18)-2.159(11)	164.8(4), 169.2	466	0	4.5	PL	33
$[Dy_3L^x{}_2(1,2\text{-}naphthol)_5]$	2.114(6)-2.170(8)	163.4(3), 167.1(2)	439	0	4.6	PL	33
$[Dy_3L^x{}_2(2,7\text{-}hydroxycoumarin)_5]$	2.132(4)-2.168(4)	163.78(15), 168.70(15)	353	0	5.15	PL	33
$[Dy(phenN6)(HL')_2]PF_6$	2.189(5), 2.145(5)	162.91(17)	300	1800	4.24	Chiral/PL	This work
$[Dy(EO5)(R\text{-}PhO)_2](R\text{-}PhO)$	2.164(2), 2.166(2)	170.11(10)	77.7	0	3.3	no	20
$[Dy(EO5\text{-}BPh_2)(R\text{-}PhO)_2]$	2.188(2), 2.198(2)	162.67(9)	51.0	600	5.9	no	20
$[Dy(EO5\text{-}BPh_2)Cl(R\text{-}PhO)]$	2.158(2)	162.39(6)	35.0	600	6.0	no	20
$[Dy(NO_3)_3(t\text{-}Bu_3PO)_2]$	2.213(7), 2.215(7)	172.6(3)	37	800/2000	6.0	no	35
$[Dy(NO_3)_3(t\text{-}Bu_3PO)_2]\cdot 0.5CH_3CN$	2.200(2), 2.211(2)<br>2.205(2), 2.212(2)	178.8(0)<br>176.8(3)	45	800/2000	5.8	no	35

[a] The references are listed in the main text.

Computational details

Complete-active-space self-consistent field (CASSCF) calculations on mononuclear eight-coordinate complex **1** (Figure S4) on the basis of single-crystal X-ray determined geometry have been carried out with OpenMolcas[S1] program package.

The basis sets for all atoms are atomic natural orbitals from the OpenMolcas ANO-RCC library: ANO-RCC-VTZP for Dy^{III}, VTZ for close N and O; VDZ for distant atoms. The calculations employed the second order Douglas-Kroll-Hess Hamiltonian, where scalar relativistic contractions were taken into account in the basis set and the spin-orbit couplings were handled separately in the restricted active space state interaction (RASSI-SO) procedure.[S2,S3] For complex **1**, active electrons in 7 active orbitals include all *f* electrons (CAS(9, 7)) for Dy^{III} in the CASSCF calculations. To exclude all the doubts, we calculated all the roots in the active space. We have mixed the maximum number of spin-free state which was possible with our hardware (all from 21 sextets, 128 from 224 quadruplets, 130 from 490 doublets) for them. SINGLE_ANISO[S4–S6] program was used to obtain the energy levels, ***g*** tensors, magnetic axes, *et al.* based on the above CASSCF/RASSI-SO calculations.

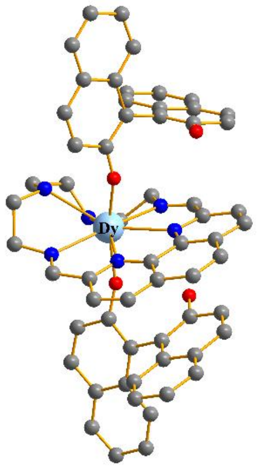

Figure S4. Molecular structure of the Cation for complex **1** Used for CASSCF Calculations; Hydrogen Atoms are Omitted for Clarify.

Table S6. Calculated Energy Levels (cm^{-1}), g (g_x, g_y, g_z) Tensors and the Predominate m_J of the Lowest Eight Kramers Doublets (KDs) of Complex **1** Using CASSCF/RASSI-SO with OpenMolcas.

KDs	E/cm^{-1}	g		m_J
0	0.0	g_x	0.002	±15/2
		g_y	0.003	
		g_z	19.857	
1	404.1	g_x	0.205	±13/2
		g_y	0.224	
		g_z	16.908	
2	692.5	g_x	0.161	-
		g_y	0.582	
		g_z	13.234	
3	823.0	g_x	4.218	-
		g_y	4.992	
		g_z	10.066	
4	880.8	g_x	0.627	-
		g_y	1.773	
		g_z	13.789	
5	909.7	g_x	0.285	-
		g_y	4.504	
		g_z	13.838	
6	978.0	g_x	0.750	-
		g_y	1.930	
		g_z	13.108	
7	1054.2	g_x	0.843	-
		g_y	2.359	
		g_z	16.574	

Table S7. Wave Functions with Definite Projection of the Total Moment | m_J > for the Lowest Eight KDs of Complex **1** Using CASSCF/RASSI-SO with OpenMolcas.

<table>
<tr><th>E/cm^{−1}</th><th>wave functions</th></tr>
<tr><td>0.0</td><td>99.7%|±15/2></td></tr>
<tr><td>404.1</td><td>75.9%|+13/2> + 22.6%|–13/2></td></tr>
<tr><td>692.5</td><td>89.4%|±11/2> + 5.8%|±1/2></td></tr>
<tr><td>823.0</td><td>42.9%|±9/2> + 32.7%|±3/2> + 8.7%|±7/2> + 8.0%|±5/2> + 6.3%|±1/2></td></tr>
<tr><td>880.8</td><td>12.9%|±9/2>+20.5%|±7/2>+26.7%|±5/2>+11.8%|±3/2>+26.1%|±1/2></td></tr>
<tr><td>909.7</td><td>7.9%|±9/2>+15.9%|±7/2>+14.7%|±5/2>+49.6%|±1/2></td></tr>
<tr><td>978.0</td><td>28.8%|±9/2>+15.0%|±7/2>+10.1%|±5/2>+37.8%|±3/2>+7.2%|±1/2></td></tr>
<tr><td>1054.2</td><td>5.9%|±9/2>+38.8%|±7/2>+38.3%|±5/2>+11.2%|±3/2></td></tr>
</table>

References:

S1 Galván, I. F.; Vacher, M.; Alavi, A.; Angeli, C.; Aquilante, F.; Autschbach, J.; Bao, J. J.; Bokarev, S. I.; Bogdanov, N. A.; Carlson, R. K.; Chibotaru, L. F.; Creutzberg, J.; Dattani, N.; Delcey, M. G.; Dong, S. S.; Dreuw, A.; Freitag, L.; Frutos, L. M.; Gagliardi, L.; Gendron, F.; Giussani, A.; González, L.; Grell, G.; Guo, M. Y.; Hoyer, C. E.; Johansson, M.; Keller, S.; Knecht, S.; Kovacevic, G.; Källman, E.; Manni, G. L.; Lundberg, M.; Ma, Y. J.; Mai, S.; Malhado, J. P.; Malmqvist, P. Å.; Marquetand, P.; Mewes, S. A.; Norell, J.; Olivucci, M.; Oppel, M.; Phung, Q. M.; Pierloot, K.; Plasser, F.; Reiher, M.; Sand, A. M.; Schapiro, I.; Sharma, P.; Stein, C. J.; Sørensen, L. K.; Truhlar, D. G.; Ugandi, M.; Ungur, L.; Valentini, A.; Vancoillie, S.; Veryazov, V.; Weser, O.; Wesołowski, T. A.; Widmark, Per-Olof.; Wouters, S.; Zech, A.; Zobel, J. P.; Lindh. R. *J. Chem. Theory Comput.* **2019**, *15*, 5925–5964.

S2 Malmqvist, P. Å.; Roos, B. O.; Schimmelpfennig, B. *Chem. Phys. Lett.* **2002**, *357,* 230–240.

S3 Heß, B. A.; Marian, C. M.; Wahlgren, U.; Gropen, O. *Chem. Phys. Lett.* **1996**, *251,* 365–371.

S4 Chibotaru, L. F.; Ungur, L.; Soncini, A. *Angew. Chem., Int. Ed.* **2008**, *47*, 4126–4129.

S5 Ungur, L.; Van den Heuvel, W.; Chibotaru, L. F. *New J. Chem.* **2009**, *33*, 1224–1230.

S6 Chibotaru, L. F.; Ungur, L.; Aronica, C.; Elmoll, H.; Pilet, G.; Luneau, D. *J. Am. Chem. Soc.* **2008**, *130*, 12445–12455.
